# On how people deal with industrialized and non-industrialized food: A theoretical analysis

**DOI:** 10.3389/fnut.2022.948262

**Published:** 2022-09-02

**Authors:** Alessandra Amorim, João Borges Laurindo, Paulo José do Amaral Sobral

**Affiliations:** ^1^Department of Food Engineering, Faculty of Animal Science and Food Engineering, University of São Paulo, Pirassununga, SP, Brazil; ^2^Department of Chemical and Food Engineering, Federal University of Santa Catarina, EQA/CTC/UFSC, Florianópolis, SC, Brazil; ^3^Food Research Center (FoRC), University of São Paulo, São Paulo, SP, Brazil

**Keywords:** food industry, industrialized food, food-anthropology, neophobia, culture, FBDG

## Abstract

“Canned, frozen, processed, ultra-processed, functional” etc. Two hundred years after the beginning of the food industry, industrialized food has evolved with many labels. Every person in the world eats and has different experiences with food that are connected to culture and social relationships which permeate our daily lives in many kinds of situations. Food evokes feelings, beliefs, desires, and moral values. For many people, food not only satisfies hunger and sustains life, but it also brings a delicious pleasure that is with their history, culture, and ancestry. Today's food industry pushes products through its marketing, which promotes a plethora of claims that have now trended proportionally with neophobic dimensions. In reality, the general public lacks objective knowledge about the complex science of modern food technology because of its low transparency, and this has resulted in the appearance of misleading ideas that can prejudice the correct analysis of food values. Given this, education about food is an urgent need. Notably, food scientists, technologists, and engineers must look at eaters through the prism of consumers who are human beings in all their rich social/anthropological diversity. The objective of this article is to explore the elemental anthropologic aspects of foods and how they can affect consumer's trust in the food industry's role.

## Introduction

Food has always played an important role in humanity's development. It was an essential element during the cognitive, agricultural, scientific, industrial, and green revolutions. Since the Cognitive Revolution (circa 70 thousand years ago), *Homo sapiens* have been able to reflect, change and transmit knowledge to future generations, molding the social, economic, relational norms and values that created cultures (1). In centuries past, especially during the Middle Ages and the colonization period, food was also an impetus for political, economic, and power upheavals (2).

Fire has been frequently and exclusively used by *Homo sapiens* for about 300 thousand years to cook foods, and it is the most ancient thermal treatment (1, 2). In the book “Sapiens, a brief history of humankind”, Harari (1) states that fire not only changed the molecular structure of food by transforming it into products easily digested, but fire also altered biology and history. The variety of foods and the shortened time to eat and digest them, which fire “cooking” made possible, could explain the larger size of the human brain (3), as well as its shorter intestine (1, 4). Fire also powered the development and the diversity of cultures (5). Omnivore feeding was transformed, and the human species went from insignificant animals to thinking beings that eventually dominated the planet and the other species, even though the *Homo sapiens* were not necessarily the physically strongest (1).

Cooking, whether at home (using fire) or in the industry (using saturated water vapor) has been one the most ingenious resources invented by civilization (1). It is the evolutionary act of manipulating and combining components to make food creations of does not exist naturally in nature, such as cheese, yogurt, sauces, pasta, cakes, etc. (2). Industrialized food and the use of heat treatments increase the period of conservation and consumption of food (6) by reducing losses and preventing diseases (7), in addition to permitting more variety and diversity in the food choices.

This thermal treatment, i.e., the binomial time-temperature, is one of the main process parameters controlled in a thermal unit operation, which are used to transform all kinds of food into edible food and beverages (meat, grains, and vegetables, coffee, tea, etc.). Sometimes, “in home” or “by industry” processes are only used to change the texture, taste, and flavor of the food, such as stewed or boiled vegetables (8). The application of this unit operation on an industrial scale is relatively recent. Indeed, wars stimulated the industrialized development of food almost 300 years ago. In the 18th century, Nicolas Appert was awarded by the French government for developing a food preserved method that allowed feeding troops during the Napoleonic Wars: The “appertization” (9–15). Some years later, also in France, Louis Pasteur realized that the method developed by Nicolas Appert (heat application) was capable of reducing the microbiological population in food (9, 10), which made food safe to consume and increased their shelf-life (that is, the time needed for food to rot) was lengthened, and consequently, food was able to be safely preserved for consumption for a longer period.

Later, Nicolas Appert's and Louis Pasteur's experiments, complemented by the studies of Peter Durand's studies in England and Raymond Chevallier Appert's (Nicolas Appert's nephew) in France, opened the way to thermal treatments such as pasteurizations and sterilizations (Table 1) (10), which are widely used today in the food industry for milk and meat products, tomatoes sauces, canned vegetables, etc. to reduce viable microorganism population into processed food.

**Table 1:** Industrial food thermal treatments.

	**Pasteurization**	**Sterilization**	**Observations**
Conventional	63–65 °C/ 30 min.	121 °C/ 21 min.	Applied with packaged solids or liquids foods. Continuous or batch process. Higher energy footprint.
High^a^ and ultra-high^b^ temperature	75 °C/ 15 sec.	145 °C/ 4–5 sec.	Applied with unpackaged liquid foods. Continuous process. Lower energy footprint.

^a^HTST, High temperature, short time; ^b^UHT, Ultra-high temperature.

Thermal treatment drastically reduced food poisonings and deaths from foodborne diseases by reducing the microorganism's population and this allowed expeditions from England to the Artic and the discovery of the Northwest Passage in 1819 (14). Moreover, Europe had a history characterized by food supply chain crises and poisonings, so the possibility of safe food storage was viewed with an enthusiasm that propelled the development of the food industry and food sciences (14, 16).

The age of the Industrial Revolution also saw the industrialization of artisanal and homemade foods on a large which allowed employee to stay a longer time outside home, including women (7, 17–19). This facilitated a revolution in the Food Industry that, in turn, facilitated the migration of rural populations to the urban centers, which propagated many lifestyle changes. With these developments, women, who were traditionally responsible for domestic services, started entering into the labor market (7, 20). Given less time for cooking at home, industry also developed labor saving adjuncts like special ingredients and convenience foods, domestic appliances, and read-to-eat food services such as restaurants (19). Although these lifestyle changes were not necessarily instilled by food industries, they did make a major contribution to support it.

During the 20th-century, food studies on the molecular level developed the knowledge of emulsion production and stability, the effect of water activity and glass transition in foods conservation, the use of bioactive compounds as food additives, hurdle technology, and new packaging systems, among others. Additionally, process innovations such as drying, extrusion, refrigeration, and freezing (10, 18, 21) were developed for products such as sauces, mayonnaise, ice cream, pasta, breakfast cereals, among many others (18, 21). According to Aguilera (18), technological improvements and molecular studies on oils, fats, sugars, protein flours, and hydrocolloids have brought many applications to domestic and industrial food processing. Many products, flavors, and textures have been created and are now consumed around the world. Eventually, macromolecules have become nutrients, and this has led to food also claiming functional roles. Furthermore, the 20th century was also marked by the discovery and development of polymers, biopolymers, and food packaging improvements. Both at the industrial and domestic levels, today's foods can be consumed many days after preparation, thanks to processing, packaging and storage technologies based on scientific knowledge generated by a huge amount of high-quality research from Food Science, Technology and Engineering.

Although similar to homemade food, restaurants have the same function as the industry: to feed people that do not want or have time to cook (19), but they do not have the shelf-life concern faced by the industry. The work routines in urban regions and the presence of restaurants (franchise or not) increased the population of those who eat outside home, which in the past was restricted mostly to workers during work time or on festive occasions. Nowadays, “eating out” is a more frequent as a leisure time enjoyed with family, friends, or alone (22). With transport development, globalization and the food industry, people can move easily to different cities and countries. Regional foods crossed oceans and were introduced into other diets. Due to technological development, food can be consumed out of season elsewhere (20). For example, Chilean grapes are found in Brazil, and tropical fruits in Europe are available year round (19). Although social and geopolitical concerns are still linked to food, thanks to the food industry and the leap in food production, eating is no longer a privilege but has become a right (20). Nowadays, there is enough food production for everyone globally (7, 20).

In light of the above, this article provides a brief critical review on food from a holistic viewpoint that reflects human consumption behavior and the eater/consumer relationship with the food supply chain (producers, food industry, and services), regulatory bodies, and political entities. Initially, the discussion will define food functionality in the sphere of relevant professional groups of Food Scientists, Technologists, and Engineers (FSTE) and those scholars responsible for the development of industrialized food. Next will be an examination of foods as social and cultural habits, followed by the different roles that foods hold for human beings, from both physiological and emotional points of view (hedonism, fearfulness, blame, and sense of security). This will include definitions of the industrial, artisanal and traditional foods in society and the understanding and acceptance of industrialized food by the eater/consumer. Finally, an additional reflection about food from mystical and symbolic points in terms of philosophies of life will be examined. It is essential to stress that each of these issues is complex and has been deeply discussed by anthropologists, sociologists, and psychologists in their domain of studies. Notably, this review has a transversal characteristic, in that the summary's focus proposes renewed direction by Food Science, Technology and Engineering (FSTE) professionals to go beyond the technical/economic points of view by focusing more on consumers as human beings. Further to this, the article ends by stating the need and importance for FSTE professionals to be included in all public health debates and classifications.

## Is it food?

Eating occurs in cultural modes (2, 5, 8, 20, 23–25), and cuisine reflects the cultural, social, symbolic, economic, and history of a population (23). Cooking is food's passage from its natural to its cultural state (5, 8, 24, 25). According to Lévi-Strauss (5, 25), culture mediates the relationship between humans and everything surrounding them. To them, the kitchen has its own language, which changes according to society. In cuisine, food is not simply prepared; it is prepared in a specific procedure or another and demands a pan, the cultural element that represents civility. The cuisine defines the human condition in all its attributes, even those that may seem “unquestionably natural” (5).

**Figure 1:**
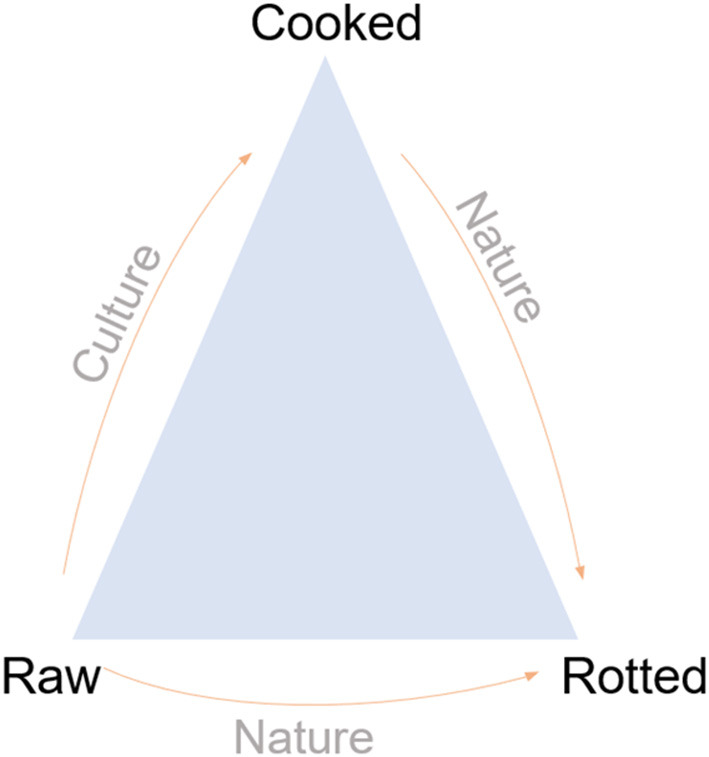
The culinary triangle. Lévi-Strauss (5, 25) with adaptations.

In the “The Culinary Triangle” (Figure 1), Lévi-Strauss (5, 25) described that nature and culture are in opposed way mediated by the kitchen. In one aspect, raw food represents nature and is connected to cooked food by the culture which, in turn, finally returns to nature in its rotten condition. This concept has been changing nowadays, for example, the appearance of vegan diets and biological (“natural”) foods. In this way, FSTE plays a role similar to that of the kitchen with a better food cooking by controlling technical parameters of unit operations, additives and packaging contributing to prolongs food shelf-life as much as possible before it returns to its rotten condition. Because of food's complexities and cultural values, this kind of change can generate identity conflicts for the eater/consumer (8).

The decision to eat is also cultural (1, 2, 26–30). As processing food to provide energy and nutrients to keep the human organism functioning, the *Homo sapiens* developed many integrated patterns of human knowledge, beliefs, and behaviors about food that are learned, shared, and transmitted across generations, transforming food in culture (26). Culture and consumption are not only interconnected but also inseparable. By helping to make sense of everything that surrounds *Homo sapiens*, culture determines and controls the criteria and distinction about what is acceptable, marketable and, therefore, capable of consumption (30). In this way, individuals eat what is allowed and accessible to their cultures (8, 31, 32). People in Asian countries eat dishes prepared with insects (Indonesia, Thailand, Filipinas, etc.) and dogs (China and Korea). Italian and French eat snail and rabbit meat, while this is not common for Brazilians and British, although England and Brazil, among others, consume products from cattle, pigs, and poultry. These and many other rejections occur mainly because of moral aspects are at work in each culture (33). The dog is a life partner to the Brazilians and British, which does not occur with cattle (29), while in India, where the cow is sacred, it cannot be slaughtered and consumed as food (3, 8).

There is also a difference between the meal and food/foodstuff. Meals are connected to culture (2, 23, 34); however, FSTE understands food from a technical point of view. Food and foodstuff are the products that we can eat, being considered as food those processed at home, and foodstuff, those processed in an industry, independently of their degree of processing (4, 18, 35). This is what our evolutionary characteristics—dentition, jaw, and bowels—allow us to eat without representing risks to our lives. Nevertheless, some kinds of food can be eaten only after being processed (at home or industry), that is, as foodstuff: rice, beans, corn, potato, and cassava are not consumed as fresh food; however, they are excellent energy sources after cooking and/or processing. Similarly, some foods such as wheat, soybean, olive, and nuts, among others, are raw materials for foodstuff, which means that they are usually eaten only after more complex processing without necessarily using additives (36). Thus, to the FSTE professionals, a meal is what we eat, and food/foodstuff is what can be transformed into meals, independently of being classified as raw material, minimally processed, processed or ultra-processed foods, according to NOVA classification (37).

The FSTE professional deeply considers the microbiology, sensory and nutritional quality of raw material, water, ingredients, and final products for development. The focus is to attend to consumer needs by providing satisfaction, pleasure, and nutrition in safe conditions (17, 38). There is also the maintenance of the consumer's quality of life, both from a health and lifestyle points of view. FSTE professionals aim to supply food to every person and all lifestyles around the world. To those who like to cook, the industry offers simple ingredients, such as salt, oils, flour, sugar, spices, etc., or more complex combinations such as emulsions, flours, sauces, meat, vegetable extracts, and milk cream, among others. On the other hand, there are convenience products for those who have practical lifestyles (4, 8, 39). With industrialization, it is estimated that 80–90% of the ingredients and food used in home cooking are at least semi-processed by industry (9, 17). All this concern intends to satisfy the consumer, who is a human being shaped by his culture, full of feelings and insecurities. However, FSTE professionals do not explore anthropologic aspects, and this sometimes results in a weak connection between food processing developers and the consumer.

Human identity is built by memories, affection, sensorial experiences, and nostalgia (34). Some groups understand food as a product of rituals and traditions materialized during the cooking act. For them, food is more than a simple meal that provides energy and nutrients to the body, rather it is a symbol of their culture, ancestry and part of their identity (20, 24, 40), or in other words, it is performed by practices and relationships that are central to social reproduction (41). Some folks still believe that the feelings experienced from the act of cooking (including the feelings experienced by the slaughtered animals) can be passed on to the food, transforming it into a “blessed” or “cursed” meal. Therefore, from the cultural point of view, food nourishes and the meal has a “soul” (20, 24, 40). To some folks, industrialized food represents a threat to their cuisine tradition and the food cultural heritage (20, 24, 34).

In the modern world, practicality can be an imposed necessity (34, 42). For some people, urbanization and industrialization have reduced the steps in cooking preparation and supplanted a pre-processed industrialized food, which has become separated from its natural origin as a commodity (20, 34). Jean-Pierre Poulain, in his book “The Sociology of Food” (20), explains that, in a modern structural context, the individual loses his role as an eater and becomes more of a consumer. Poulain also describes that the food industry has its roots in the familial cooking space, attacking its socializing function, without assuming it. Food and cuisine are elements of collective feelings and belongings (8); although it is technically incorrect and without scientific evidence, it is possible to understand the origin of some expressions like “real food” to mean home processed food. These identity groups have difficulty accepting the inclusion of industrialized food in society due to their moral values and affective memories, which are rooted in their culture (20).

Food is identity (2, 20). It is even possible to recognize the individual's personality traits throughout the elements that permeate their diet (24). The cuisine is the last aspect that changed during the assimilation process (8). FSTE professionals and the Food Industry as a whole aim to attend to the food demand of *Homo sapiens* with diversity. In this way, FSTE professionals should thoroughly understand the cultural aspects that permeate the eater/customer. In reality, the human being does not feed on complex molecules; most people feed on habits, rituals, knowledge, and sensations that this food represents (32). According to Claude Lévi-Strauss, food is not “good only to eat but is also good to think” (24).

## Food, culture, and social interaction

Eating is a way to communicate, and it is part of social relationships (5, 22, 23, 29, 31). The act of eating together with others is typical behavior of *Homo sapiens*. The human being does not come together to eat and drink but to drink and eat together, socially, in an interaction act (2). Eating is a complex phenomenon that includes biological, psychological and social aspects (8, 20, 24, 40, 43). More than a physiological need, food is associated with a sociocultural folk's identity (3, 24, 29, 31, 40, 44). Folk cuisine originates from a historical process and is loaded with singular traditions that, as belonging to a dynamic society, are constantly transforming and changing (44, 45).

There is a distinction between eating (social action) and nourishing (biologic act) (46). Eating preference is not individual, and it is associated mainly with cultural aspects (8, 24, 29, 40). Food consumption, in addition to nutritional requirements, is influenced by hedonism, moral responsibility, convenience situations (such as vacations, parties, and celebrations) (40, 43, 47, 48) and lifestyle (likes, working/study hours, leisure time to shop, cook, eat and do the household chores) (23, 24, 48).

Eating is part of many temporal cycles, whether related to obtaining food (planting, harvesting, production, and availability). It can be fasting (characterized by the absence of food) or festive (when a lot of foods are allowed) (20). These biological and social aspects are marked by many interactions. Eating is the first step in human social learning (29), which evolves into more complex human relationships. Friendships, neighbor relations, and even politics also revolve around food (22, 40). Sharing the meal, especially at home, is the first phase of the group association (2). In childhood, as biological mechanisms emerge they are modulated by these social aspects (breastfeeding, rest and work parent's time). As the child starts eating food in replacement of breast milk, the biological and the social merge to culturally adapt (20, 40).

The habits learned during childhood are modified throughout life, primarily as the outcomes of social interactions experienced at school and in professional environments, when the personal identity and the sense of belonging are formed (24). From the Latin *habitus*, habit means constant willingness to act in a certain way (46). Thus, eating habits represent a contextualized attitude that is regularly and unconsciously repeated and results in an acquired disposition associated with psychological and social meanings, which are difficult to modify after acquisition (33, 49). Conversely, food preferences have been transformed into habits and traditions over the centuries, and time is needed to modify them (3, 31).

With industrial developments and the consequent urbanization processes, society has become less dependent on the harvest cycle (2). The concentration in urban centers changed the food trade and people's relationships over the time (2, 29). Today, products are sold at supermarkets (10, 20, 42) and their prices carry intrinsic value quantified in money. The barter and exchange systems no longer exist. Food is now stored in refrigerators (10), and not preserved in animal fat, salt, or vinegar (2). Time is no longer measured by the sun's movement, and food access is no longer directly dependent on the growth of plants and animals (29). Clocks have become essential (1). With stipulated times to start and stop, the workforce is now rewarded with money instead of actual goods for sustenance. The week has been divided into workdays and days off (1). Women, the traditional keepers of food knowledge and responsibilities for cooking, joined the labor market (20, 42). Communities and families were replaced by the state and markets and religiosity by secularism (1).

The evolution of civilization has also changed cuisine habits (20). The floor fire and simple stove have been transformed into gas or electric appliances, which takes less time to cook (50). To protect food and reduce waste nowadays, food is sold inside packaging and frozen in freezers (10), rather than displayed in blocks of snow, fat, or brine (4). Products and regional ingredients have crossed over the geographic barriers (19). With globalization, some cuisine traditions disappeared while others expanded, created, or “fused” in modern terms. For example, potatoes were included in Irish cuisine, tomatoes in North-American, corn and cassava in Africa and Europe, wheat flour in Brazil (29), and Mexican pepper in India (1).

Rising from different geographic cultures, foods have hardly kept their original characteristics (2). For example, a sweet drink produced in Switzerland by a local company, if marketed in France, will have the sweetness reduced. In the same way, if the target audience of this company is Italian, Portuguese, or Brazilian, the sugar content probably will be higher than the original one (2). These cultural adaptations can also be exemplified by the coffee that, even from the same brand, has a different flavor in Italy, Denmark, and USA (20). No matter the processing place (industry, home, or franchise restaurants), food will undergo modifications based on the contemporary food habits of where it is eaten. In France, McDonald's franchises offer beer as a drink option; in the USA and Brazil, only non-alcoholic beverages or soft drinks are options. In France, Netherlands, and Belgium, fries are accompanied by mayonnaise, while in the USA it is ketchup, but in Brazil it is both mayonnaise and ketchup, whereas in Quebec (Canada), a sauce and cheese, similar to poutine (20) is popular.

Poulain (20), as well as Fischler (24) and Montanari (2), considered that globalization and the market's internationalization will result in culinary compositions and re-compositions; therefore, globalization is not restricted in being a destructive source of regional food and culture. Industrialized food has no symbolic, moral, or ideological value as traditions. Nonetheless, even inside the same culture it is possible to have differences, such as the definitions of the food, the way it is processed, the rules for eating, and even the attached moral values. Thus, besides it being on the stage with symbolic and ideological conflicts, food also identifies boundaries in distinct cultures (20). In this way, culinary traditions cannot be simplified to ingredients or recipes fixed to some place or time (40, 44).

To Contreras and Ribas (51), our omnivore deculturization will happen due to food's medicalization, and not only because of food industrialization. The belief that health can be attained just by food choices will transform food into healthy molecules that prevent illness. It is well known that the low consumption of nutritious foods can cause diseases; thus, food can be considered as a source of health.

## Physiology, hedonism, fearfulness, and blame

The primary function of food is to supply energy and nutrients for the maintenance of life. The human being eats to live (31). By definition, diet is the individual's dietary pattern (52). It is a source of health, taste and pleasure and is influenced by culture, geographical localization, religion, and lifestyle (52). On the other hand, when inadequate, diet can be also a source of illness (7, 8). Despite increases in food production, people are still hungry, malnourished, and overweight (7, 9, 53–55). Malnourishment and obesity are reflexes of inefficient or wrong food intake, unbalanced by nutritional and caloric points (7, 34, 53). Access to nourishing food is essential to providing the physiological needs of humans and maintaining life; however, the lack of education about food hampers good health (7, 53). In this way, fake news and misinformation can create insecurities and uncertainties related to food intake and may induce anxiety and even cause panic situations (20).

*Homo sapiens* have not yet completely learned to control their brains, their desires nor their reactions (56). When neurons are activated and synapses fire unconsciously, they produce biochemical processes that have been influenced by cultural factors. Desires are not planned; we just feel them. In this context, the external and virtual world—many times unreal—can cause significant damage, such as an obsessive search for opinions, feelings, and desires, which are manifested in the need for social belonging (28, 56). The relation between hunger-satiety is also influenced by hedonism (29), and the exaggerated concern with diets can cause psychological unbalance, a decreased quality of life, and lower life expectancy (33). In other words, by provoking anxiety in the eater, exacerbated concerns about diet can harm health rather than improve it. For example, North Americans are generally more concerned about diet than the French (especially about health and appearance); however, the French have a healthier diet than North Americans (8, 33, 57, 58). On the other hand, in a recent cross-cultural study, Sproesser et al. (59) analyze 10 countries (Brazil, China, France, Germany, Ghana, India, Japan, Mexico, Turkey, and the USA) with regard to traditional and modern eating, and in contrast to past studies (33, 60, 61), attitudes to food or potion sizes when it comes to what constitutes traditional and modern eating, USA and France, now appears similar. Additionally, Sproesser et al. (59) also describe that in countries with huge extension (such as Brazil and USA) probably there might be heterogeneity not only in terms of different regions but also with regard to different ethnic groups within one country.

Guiding food choices, as presented in the Food-Based Dietary Guidelines (7) by food classification strategies and considerations of food-intake behavior, is extremely complex (62). In addition to accessibility, availability, taste, nutrition, or the consumption situation (such as festive or daily one), there are also emotional, cognitive, psychosocial, and cultural issues (8, 24, 48). Food choices are specific to the context. The social environment is an essential delimiter of likes and choices (32). Social life is modulated by feelings and definitions of what is allowed/prohibited and even from what is impure (8, 31). Impurity is related to blame, gluttony, disgust, and laziness. Gluttony is associated with pleasure in eating. Laziness is a certain discouragement to daily cooking, which can be understood as an aversion to work. Blame and disgust are about whether or not the food is good to eat, but in a cultural judgment, there is no relation with health (31, 46). Food is frequently consumed in moral terms due to what the cultural conceptualization regards as good and bad (acceptable/not acceptable), not necessarily or exclusively, taking into account particular likes of individuals such as the taste of the food or even the desire to eat it (2). In this way, a food transgression can imply moral judgment and blame in the eater (8). Blame is also linked to the food ingredients, which can be understood as dangerous to eaters (31).

These feelings cause conflicts to the eater that can harm their physical and mental health. In the contemporary world, hedonism has been assumed an emotional rather than a sensory character (30). Most healthy foods are not tasty. In this context, the desire for healthy-eating opposes hedonism. Fresh food is seen as pure, while industrialized food is viewed as artificial (6, 47, 63–66). Recently, psychologists defined “*orthorexia nervosa*” as the obsession to eat healthy (67, 68). According to Bhattacharya et al. (69), *orthorexia nervosa* describes a fixation on food purity involving ritualized eating patterns and a rigid avoidance of unhealthy foods. Unlike anorexia and bulimia nervosa, orthorexia is related to food quality (in a healthy sense) and not quantity or corporal mass (68). Watchful to the market, some brands are offering food products that meet these customer's needs (31), including rescuing the idea of nostalgia and tradition (70). Nonetheless, cultural and emotional rescue involves the use of terminologies and definitions that are not yet clearly defined, such as artisanal, traditional, and natural food, which are being specially labeled by food producers (companies or entrepreneurial enterprises) and which can carry can mistakes and misinformation that consequently engender more insecurity, distrust, and anxiety in the eater. Because of this, transparency is fundamental for food industries (10).

## Food industry, traditional recipe, and fast food

Full of ancestry, many cuisines have been changing over centuries. Even in places famous for their traditional heritage, it is hard to find meals with the same taste that were made by past generations. Tradition is mutable; however, the meals carry worldviews (71). If one recipe dies, it will take its vision (2, 71). In the modern and globalized world, food preference is divided between the traditional (cultural heritage) and the modern (international, innovative, and practical) (72, 73). Products never seen or tried by some cultures have started to appear on supermarket's shelves, restaurants, food events, and over the years, frequently inside homes (8, 20). Avocado, guacamole, kiwi fruit, tabbouleh, paella, tacos, pizzas, pineapple, soy source, raw fish, among others regional culture dishes, are present worldwide nowadays in many cultures (8).

Montanari (2) describes how *Homo sapiens* used agriculture to build food-induced post-industrial cultures into a mistaken conclusion that there is fundamental naturality in agrarian activities, usually considered as tradition. There is no definition of natural products (64–66). For example, flour obtained from wheat—present naturally in nature—gives rise to bread that, in turn, does not exist naturally in nature and yet is considered a traditional food in several countries of the world. The same can be considered with the cheeses, wines, and beers of French, Italians, and Germans, respectively. In addition, there is also a mistaken understanding that “more natural” foods are safer (9). This kind of thinking ignores that toxins and pathogens extremely dangerous to life can be naturally present in fresh foods. To Montanari (2), the differentiation between what is naturally in nature and what is obtained from it distinguished human and animal identities and, from the social point of view, originated civilization.

Fischler (8, 42) reviewed some historical changes in cuisine. In the last century, circa the 1930's, a considerable amount of collective culinary activity was redirected from the kitchen to industry (42). In the past, cuisine knowledge was transmitted essentially from mother to daughter (8). With the functional social changes of urbanization and the advance of industrialization processes, many women entered the workforce. The role of cooking and the perpetuation of cooking knowledge were no longer exclusive to women to teach and learn. Recently, although in lesser numbers, men also have been working in the kitchen (7, 8, 42). Nowadays, food knowledge (traditional or not) can also be obtained individually, by books, videos or from social relationships that do not necessarily involve family or other feminine authority (8).

This reality especially challenges the traditional cuisine producers that, depending on the customer acceptance, have to make minor changes in the recipes to improve health, safety and convenience (74) without losing the tradition and taste. Currently, health issues can overlap the traditional issues (73). Souza Junior (71) relates that in the Candomblé religion, where tradition is valued, it is possible to note the incorporation of industrial ingredients and the rejection of the traditional ones to avoid illness. Although understood as healthier by the lay population, there is no correlation between healthiness, traditional food (73), and industrialized food (75). The Mediterranean diet is considered healthy by the scientific community (76); however, traditional products consumed by these peoples, such as hams, olives, pastries, and cheeses, can have high contents of salt and/or fats (77), as a percentage of energy, total fat content can be as high as 40% with over half being monounsaturated fat (76). Even so, some of them have been classified as ultra-processed foods, which means unhealthy in some Food-Based Dietary Guideline (FBDG), such as Brazil's, which uses the NOVA classification (78). The Mediterranean diet is healthy because of its nutritional biodiversity and moderate consumption, complemented by philosophy of life that values personal relationships, the pursuit of happiness and physical activity (73, 79), and not necessarily in the function of the quantity of unit operations that food has been submitted. The Mediterranean diet pyramid has socio-cultural relationships and physical activities on its base, i.e., as a priority even before food choices (79). The Brazilian FBDG, despite using NOVA classification, also orientates people to experience social and pleasurable eating time.

Traditional food is made with regular ingredients, following the usual processes of traditional recipes. The tradition involves knowledge, techniques, transmitted values (2), and emotional and ancestral issues (73). There is no official definition of traditional food. Guerrero et al. (74) explained that traditional food can be “a product frequently consumed or associated with specific celebrations and/or seasons. It is normally transmitted from one generation to another, made accurately in a specific way according to the gastronomic heritage, with little or no processing/manipulation, distinguished, and known because of its sensory properties and associated with a certain local area, region, or country”. According to the European Commission “traditional means proven usage in the community market for a period showing transmission between generations; this period should be the one generally ascribed as one human generation, at least 25 years”. Readers interested in studying the definitions of traditional food are invited to consult Guerrero et al. (74).

Tradition is part of the food's cultural heritage (80); however, culture is related to tradition and innovation (2). Nonetheless, in the contemporary world—practical, international and industrialized—is it possible to have the same food as our ancestors, even by a traditional recipe? Ingredients are everything that is incorporated into a recipe (72). Nowadays, to guarantee food safety, the ingredients have been industrialized. Regardless of the safety issues, could modern ingredients modify a traditional dish? Reconstructing the original recipe is highly ambitious (2). Despite the ingredients, could modernity, viable by domestic utensils (stove, steel or aluminum pans etc.), modify traditional dishes? Cooking is a skill of combinations (2) that, over the years, can proportionate new dishes or newly adapted versions of dishes (2, 20, 24). As with culture, human taste is not static (2); therefore, the perception of different flavors of traditional dishes can be due to the modification of ingredients, preparation method and taste. In addition, according to Montanari (2), the human organ responsible for the perception of taste is the brain, and not the tongue, and the brain's perception, in turn, is strongly influenced by our culture.

Another diet consequence of the modern lifestyle involves time. Stimulated by the accelerated routine and often full of anxiety, people choose food that does not require more time and stress in their decision-making. In this context, fast-food chains have increased worldwide as business model franchises, such as McDonald's, Subway, Starbucks, KFC, Taco Bell, Domino's, Pizza Hut, Dunkin Donuts, Papa John's, Burger King etc. Fast food offers convenience with little tradition (8, 42), and other similar franchise-type restaurants now dominate food plazas of modern malls or shopping centers worldwide. This eating style induces people to have meals unconsciously, occasionally alone, to supply their physiological need (hunger). Fast food can trigger “disenchantment with the world” and is defined by sociologists as loss of meaning and devaluation of emotion (72). In addition, the worldwide spread of this North American culture, especially in European countries, has provoked some anxiety and fear of losing national or local identity (42, 80). Generally, fast food is eaten with the fingers and without a plate or cutlery, in contraposition to other styles like the French eating etiquette or Asian traditions where a much different set of dining manners are civilized standards. This difference in the manners of eating, independent of what kind of food, can cause a conflict of feelings and moral judgments in the eater (20). Despite being associated with hamburgers and junk foods, this restaurant style provides different kinds of food, such as pizza, national food (Japanese, Korean, Mexican, Arabic, Brazilian etc.), and also traditional homemade like food.

**Figure 2:**
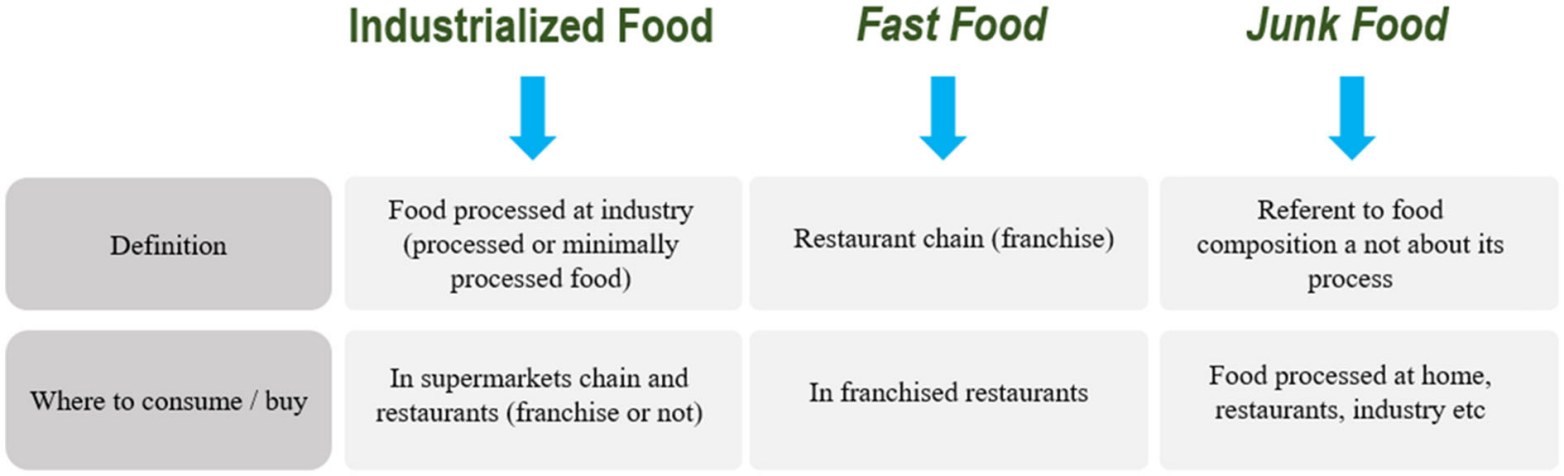
Fast food, junk food, and industrialized food—definitions and differences.

In the context of health, more than 1/3 of the worldwide dietary guidelines advise to avoid fast foods (81), but herein lies common conceptual mistakes that lump together fast food, industrialized food and junk food. Industrialized food is processed by a company with industrial equipment at an industrial level. Industrial food is available to the eater/customer by the retail segment and restaurants as well. Fast food is not necessarily industrialized food, although they can use industrialized products for cooking and an industrial philosophy to operate (similar to Fordism) (8). Further, junk food has come to signify low nutritional quality foods (82, 83), which may include food processed at industry, home, or restaurants (franchise or not). In a more accurate summary: Junk food depends on the nutritional composition of food; fast food is the restaurant's style, and industrialized food is food that is mass processed by industry (Figure 2).

For people who regard traditional foods and moral values as important, industrialized food and fast food are transgressions (20). Nevertheless, one food can be beneficial where another is not, depending on the context. Diet food is healthier for people who suffer with diabetes, but not necessarily to all the population. Regular yogurt can be good for people who do not suffer from lactose intolerance. Fish is good for people who appreciate its taste. Therefore, when food is involved, there is no universal rule. In this way, generalizations are equivalent to misinformation. Sanitary rules—such as the use of pasteurized milk to process all kinds of cheese and derivatives in some countries and a public health policy to avoid foodborne disease—affect the moral and cultural value of food. Cultural heritage and food safety are important to society and contribute to the economy (74). Public health agencies and scholars must find a way to conciliate it. In this context, the Food-Based Dietary Guidelines (FBDG) can be a powerful tool to guide food choices, exploring the country's food and culture diversity, including regionalities, beliefs, and philosophies of life, lifestyles, age group, different identities inside some culture (such as indigenous people), different conditions of life (such as breastfeeding, intolerants and allergic, etc.) among others. This, however, requires more multi and trans disciplinary work.

## Should I eat it?

To Fischler (8, 24) and Contreras (84), the omnivore experienced dilemmas that the cow or koala never had. *Homo sapiens* have a vast variety of foods, taboos, rules, traditions, and beliefs, resulting in conflicting emotions, mainly about the unknown. Neophobia and neophilia are conflicts experienced by humans when faced with an unknown food (47). Neophobia is the fear and rejection of the new, while neophilia is the fear and curiosity about the unknown (8, 24, 29, 47, 85, 86). In contrast with domestically processed foods, industrialized food causes more rejection and unsafe feelings in eaters (85).

When faced with industrialized food, the eater/customer does not know the origin, the quality, and the history of the food (8, 24, 51). Therefore, food processed at home and a part of the country's culture produces less neophobia and brings tranquility and mainly familiarity to the eater (34, 47, 85). Industry must inform and be clear about the new product's ingredients and consider their risks and benefits to reduce neophobia and improve eater/customer acceptability (85). With the development of the food industry, from the historical point of view, food security, food safety, and poisonings were controlled and strongly reduced (29). Scientific knowledge about microorganisms, pathogens, and toxins has never been as precise or complete as today. However, despite safety improvements, there is a mistaken perception of risk by the eater/consumer (18, 29, 70). Although food safety is one of the FSTE professionals' pillars; nonetheless, this concern is not noticed by the consumer.

The insecure feeling proportionated by the lack of this knowledge induces people to look for a food that they believe to be safer and healthier (31) as well as to idealize the past (70). Consequently, many entrepreneurs—and even big companies—have emerged selling artisanal or gourmet products that attempt to keep and rescue the traditions and origins (40). Yet, fresh products (fruits, vegetables, and animals—dairy and meat) can be a source of contaminants and diseases (39, 87). To ensure food safety in the industry, technical knowledge and good practices (such as efficient hazard analysis and critical control points—HACCP) and health regulations are primarily used (87, 88). Consumption of food, which has been erroneously deliberated for production, can ignite illnesses already controlled that are caused by viruses, bacteria, fungi, and toxins (87). In this context, especially for fresh food, minimally processed food (MPF), non-thermal processes and special active packaging have become effective optional methods to offer safe and fresh products (89).

Some literature states that the concept of risk changes according to the culture and history of the population (90, 91). Usually for French and Spanish women, pesticides, medicines, microbial contaminations, pollutants, genetically modified organisms (GMO), and epidemics represent a health risk, but these concerns for Brazilian's women are dependent on their social class (90). Industrialized food and chemical components (including food additives) also cause mistrust (29). The chemical products used by the food industry are regulated and monitored by oversight agencies of each country. For many people, however, the government sometimes seems to protect companies (agribusiness, industry, and supply chain) more than the eater/customers (29, 34). Disoriented, the consumers then only access media information, which can sometimes exacerbate fears and phobias (31, 40). Nowadays, fake news and many possible problems are exaggerated by social media interventions (7).

By definition, “a risk” is a possible future adverse effect resulting from human choices and actions (31, 49). Nonetheless, sometimes, the risk is not associated with health. For some, the risk of getting fat is related to belonging to an aesthetic standard and not only to avoid diabetes or obesity (8, 29). However, exaggerated concerns with diet, aesthetics, and fads can trigger diseases such as anorexia, bulimia (8, 53), and orthorexia nervosa (67, 68). Within the same culture, the understanding of risk can vary according to gender, social position, values, and beliefs (34, 91). Regardless of the concept, the eater/customer better accepted old or already known risks (47). Frozen foods were not well accepted by the population at the beginning of the 1940's, when the freezers started to be useful in society. Now they are commonplace (29).

The consumption decision is according to the balance between the risk perception and the perception of the product's potential benefits (91). The eater/customer feels insecure because they feel forsaken and are no longer willing to trust. Despair, skepticism, and doubt surround the eater during the decision-making (31). Purchase decisions are driven by three motivations: sensory attractiveness, biophysiological and social benefits (prestige and nutrition), and ethics (origin and ideological issues) (32). Barbosa and Campbell (30) describe that consumption and identity are linked; however, the identity is more connected to the consumer's reactions to a product (feelings and desires rather than necessity) than to the product itself. To Galindo and Portilho (31), it is inaccurate to relate purchase and trust. The purchase represents daily experimentations, permeated or not, by luck. This mistrust results in fear, which can be fed by facts or fake news (31). When a person is scared, rational human capacity is limited (29).

Consumer goods are a visible part of the culture (30, 92). Portilho (92) explains that consumption choices are related to belonging experiences that, in some cases, classify the decision made as superior or correct. In this way, consumption and culture are linked to cultural and moral aspects (93). Moreover, consumption is also associated with moral feelings such as “good citizens” or “good parents” and “good family” (94). Industry and the kitchen have the same primary function of processing and preserving foods; however, to some people food processed at home is like the “good mother”, purified by the love and familiar ritual, while industrialized food is like the “bad mother” and, therefore, a product of untrustworthy manipulations (8).

Moreover, the act of following collective thinking, especially when influenced by concepts of equality, citizenship, and freedom of thought, are the way to achieve “good, fair and happy life” (93). In this way, the understanding of food as nature leads to its idealization, which contrasts with the way most people consider some technologies and even cultural practices. This influence is a new conceptualization of what is good, healthy, and faithful (94). Food is the convergence point of state, corporations, and individuals (94). Distrust of public institutions increases the politicization of consumption (95), in which the individual perceives their consumption as a form of participation in the public sphere to boycot or “buycot” products and brands (93, 94). Currently, the customer has migrated into more critical, autonomous, and active behaviors (93, 94). Modern consumers assign responsibilities and duties to themselves in the social and environmental context (92, 93). Consequently, during 2010–2017 around 30,000 products introduced ethical, social, and environmental practices on their labeling (9).

Despite FSTE concerns about food safety, the feeling of security does not necessarily convince the customer. Although scientific knowledge has never been as voluminous as it is today (29), the concept of risk has never been so mistaken (70). The lack of knowledge about the origin, the process, and the food in general, including the controversial information advertised in the arenas of foods and the traditional and social media, fuels mistrust and moral conflicts. For the eater/customer, the right to access quality food includes the right to make free and well-informed choices, according to each individual's preference (80); therefore, transparency among institutions, eaters/consumers, and corporations becomes a vital factor in contemporary feeding (94).

## “Canned”, “ultra-processed,” and “functional” food. What do customers understand by industrialized food?

There is no life without food. Regardless of how food is understood, every person in the world eats and has at least a minimum knowledge about food (2). Before the Industrial Revolution, laypeople cultivated food without technical regulation and agency monitoring. In 1850, 90% of the population were landsmen (28), nowadays it is <40% (96). In previous eras, food poisoning and hunger were recurrent and responsible for many deaths, especially in Europe (16) and Russia (14), and were neglected in other countries. Foodborne disease and hunger began to be controlled with the development of the food industry when the thermal process was developed and applied by industry (10–14, 89). During wars, the first people to experience neophobia/neophilia with industrialized food (commercially sterilized food in glass or tin packaging) were soldiers and expeditionary troops (14).

Processing turns agricultural commodities into edible, safe, healthy, and nourishing products (97). Processing food in current industry guarantees a standardized, transportable and safe product to consume for a longer period (4, 15, 36, 39, 89). However, food acceptance of industrial and later frozen food was slow and surrounded by mistrust. To Cascudo (3), “the food industry reduces the kitchen to a cabinet with cans, where the essential technique is to open the can without hurting the fingers.” For Giralmo Sineri, “Canning is anxiety in its absolute state” (2). In addition, widespread speculations without evidence about botulism and chemical contaminants added to food at packaging had intimidated the population to consume it (14). Moreover, despite some canned food being nourishing, the perceived health loss during the thermal treatment^1^ raised neophobia (13, 16). Currently, commercially sterilized food is widely presented in the market (98); however, now it is not only canned, but also in polymer-based pouches, cardboard-based packages, and glass bottles, as well (13). To be accepted, new foods must be part of the population's habits, have good quality, an affordable price (16), and a short cooking time. It takes a long time to achieve consumer/eater trust and break down the neophilia barrier (29).

With the rise of the food industry and despite the diversity of products and packaging, all industrialized food was labeled as “canned” food. Nowadays, terms such as “processed” or “ultra-processed” food are used to mean industrialized food, both with a pejorative meaning (4, 99). However, food processed by industry is nothing more than an adaptation on a large scale of home processed food, and it is made with scientific knowledge and rigorous control (18, 35). Meals made at home or restaurants are also processed, but not always with technical control. Fortunately, they are usually consumed just after cooking, which means their shelf-life is not a concern.

The Brazilian and the Uruguayan Food-Based Dietary Guidelines (FBDG), adopted by governors as a public policy tool, classified food by their processing level to indicate nourishment (7). The term “ultra-processed” (UP) food (created by NOVA classification), means “not real” food (37) and, despite being classified by processing level, the arguments used for avoiding this food are their ingredients and not their process parameters (4, 7, 11, 15, 100). Despite the good intention behind this classification system, and most notably, there is no relation between healthiness and processing levels (75, 101). Among those foods classified as UP food, nourishing foods are included (102, 103). Moreover, diets without UP food can also be unhealthy (82), and there is still confusion about junk food definitions.

Furthermore, the term UP does not exist in Process Engineering terminologies. To the FSTE, a process is a sequence of unit operations (7), and “ultra” means high intensity–such as ultra-high temperature, ultra-filtration, filling ultra-clean, and ultra-efficient, etc.–and not quantity. The NOVA classification was created by health professionals, who are experts in health segments, such as epidemiology, and recognized inside the scientific field; however, they lack expertise in food processing (e.g., unit operations and process engineering). The terms UP and “real food” are misleading (4, 102) and do not help to improve the understanding of healthy food (7). Although the concept of UP foods has certainly entered the consumer consciousness, some mistakes have been made to unequivocally and accurately classify them, as observed by Braesco et al. (104).

Still, despite being an industrialized food, functional food has good customer acceptance and is a market trend (105). With a healthy role, functional food provides additional nutritional benefits (86, 91). Dominated by probiotic products and functional ingredients that have been developed in all food categories since the 1980's, such as dairy, soft drinks, baked goods, baby-food markets, etc. (105). People have accepted that functional food consumption improves health. Thus, despite the fact that food decision-making is intrinsically related to the historical, social, and cultural context of each country, the association of food and health has disseminated worldwide (90).

In the modern world, people are concerned about health and longevity. At the same time, convenience is a need, and the Food Industry is essential to accomplish it (9). Yet, after about 200 years of the food industry's existence and 60 years after Food Engineering became an established field of science, this has not been enough for some people to trust and feel safe with industrialized food. It is a consensus that, if safe from the microbiology and toxicologic point of view, fresh food or minimally processed food should be the main source of nutritious food, but for people who do not can foods or do not want to cook, a quality alternative must exist (103). Furthermore, people lack knowledge about industrialized food, quality, and food safety in general (9), so how can they trust in something they do not know sufficiently? The inclusion of food subjects in basic education, such as food education, food safety, nourishment, good domestic food handling, and sustainability issues, must be considered in a public policy tool (106).

## Further considerations

Some life philosophies aligned to faith understand food as a source of life or contamination. From the religious point of view, food—especially the ones related to rituals—can have spiritual meaning in addition to its nutritional value. For example, Easter eggs represent a new life and resurrection in Christ in Catholicism. The bitter herbs and bread used by Jews on Passover symbolize their periods of slavery and escape from Egypt. Moreover, in their New Year celebration and in a wish for the new year to be sweet, Jews eat honey to be fertile; eat fish to always move on and ahead, and they eat pomegranate seeds so that their good actions are multiplied (56). In the yam (or pestle) celebration of the Egibô kingdom in Nigeria, the cake preparation and consumption represent their survival and splendor, and it signifies means life and death, hunger and abundance, disease and health (Candomblé, an Afro-Brazilian religion). The elements of this ritual are synonymous with strength (71). Furthermore, to Muslim's food can influence the soul, behavior, and moral and physical health; thus, food consumed by them must be Halal, i.e., according to the law of Islam (107). According to Fambras (108) and Jia and Chaozhi (109), Halal products increase between 15-20% a year worldwide, and it is estimated that the Islam population will represent around 30% of the world population in 2050.

According to Junior Souza (71), to Afro-Brazilian religions, especially to Candomblé, food is a synonym of “axé”, which means life. To Candomblé, nothing can remain without food, and their correct consumption is related to health maintenance. Food is the source of axé and transmits vitality and heat. When the heat is over, the body dies. In addition, the rituals involved in the food preparation also are important and, if it is performed in an inappropriate way, it could provoke the opposite effect (71). Similarly, in a deep way, food is mystic for Catholicism and represents God. It is God in the mouth. Throughout the ritual, bread and wine become the body and blood of Christ (20, 40, 56).

Besides religions, food is also the center of some philosophies of life, such as vegetarianism and its derivations (veganism, flexitarianism, and others) (110–113). These derivations are a consequence of a vast eating lifestyle which either does not include or restricts the consumption of animal food (meat, eggs, milk, cheese and so on) (110). This action is motivated by ethical issues about animal well-being, the environment, and health (110, 111, 113, 114). Vegetarianism and its derivations are related to identity issues and the individual's personality (111). It is a food intake and lifestyle choice practiced by adults (113). People become vegetarian during adolescence or adulthood. Adhering to this philosophy is a conscious decision, not an imposition (111).

Although vegetarianism philosophy is old, scientific studies about its social, ecological, and health consequences are quite recent and need further deepening (110–113). Some supporters of this philosophy report losing weight with a diet without meat. Others consider that this diet can improve health and avoid diseases such as diabetes and hypertension. Furthermore, in comparison to omnivores, vegetarians usually are more concerned about health issues (111, 112). No scientific evidence, however, exists to classify vegetarianism as healthier or unhealthier feeding systems (112). The only scientific evidence is about the vitamin B12, zinc and iron absences (110).

Philosophies of life are connected to sociocultural issues and identity groups (111). In a multicultural society, all the (food) lifestyles have to be accepted and have space in society. In addition, the ideological movements related to food, besides being an arena of ethical, ecological, and public health discussion, can represent an essential role in the economy. This is a new market to be served generating new business and creating an improved economy. Time to cook and difficulty to find a convenient vegetarian food or vegetarian restaurant are the main barriers described by vegetarians (112).

As new business opportunities open, the food market tries to adapt to new demands, both in terms of operating procedures and in the development of new products. FSTE professionals are looking to develop products similar to meet with no animal sourcing. In addition, technologies such as nutritional enriching by nano or microencapsulation have been studied and applied in new products to mitigate possible nutrition losses (115). The FSTE professionals understand that healthy and sustainable food intake is a universal right regardless of religion and philosophy of life.

## Concluding remarks

Food Science, Technology, and Engineering aim to supply quality food to every single person worldwide. Quality is synonymous with safety, nourishment and taste to the professionals in these domains; however, in addition to technical and food safety knowledge, understanding social anthropology is crucial to develop and supply food quality. Eating is a complex and multifactorial issue. A multidisciplinary task is required to have success in reaching this goal. Recently, new issues about healthiness have emerged in society. Food-Based Dietary Guidelines were made worldwide to improve health and quality of life by food-intake and food choices. Nonetheless, the professionals responsible for developing food were not included in this debate, so it is not yet a complete or accurate guideline.

To be sure, an egregious conceptual mistake about processing terminologies has been made in the development and use of misleading NOVA food classifications, and these are provoking misinformation and misunderstandings. Practicality is a necessity imposed nowadays. In a dynamic multicultural society, it is impossible to live without the industry presence and accurate scientific technologies to maintain them. Unfortunately, the love of the cooking act is not enough to destroy microorganisms and toxins; unit operations are required. There is no way to move back in society's evolution and change this reality. The FSTE professionals and the food industry are now challenged to reinvent themselves by considering social drivers. Such achievement requires that all the food industry professionals and public policies developers must focus more on the anthropological perspective. Besides its physiological role, food is also an arena of feelings, insecurities, beliefs, and political actions. To improve health, understanding and treat the consumer as a human being is also essential. To be sure, FSTE has substantial scientific knowledge to help industries to guarantee high standard of quality for processed foods.

## Author contributions

AA: bibliographic investigations, writing—original draft, and writing—review and editing. JL: writing—original draft and writing—review and editing. PS: writing—review and editing and supervising. All authors contributed to the article and approved the submitted version.

## Funding

This work was funded The São Paulo State Research Foundation (FAPESP) for the grant (2013/07914-8) and the Brazilian National Council (CNPq) and for Scientific and Technological Development (CNPq) for research fellowship of PS (30.0799/2013-6). This study was financed in part by the Coordenação de Aperfeiçoamento de Pessoal de Nível Superior – Brasil (CAPES) – Finance Code 001 (M.S. fellowship of AA).

## Conflict of interest

The authors declare that the research was conducted in the absence of any commercial or financial relationships that could be construed as a potential conflict of interest.

## Publisher's note

All claims expressed in this article are solely those of the authors and do not necessarily represent those of their affiliated organizations, or those of the publisher, the editors and the reviewers. Any product that may be evaluated in this article, or claim that may be made by its manufacturer, is not guaranteed or endorsed by the publisher.

## Footnotes

^1^It is known that high temperatures can change chemical conformation of food components. Thus, the Science, Technology and Food Engineering deeply studies the binomial time-temperature with the purpose to guarantee food without microbiological risks with a less possible nutrition component losses. Fortunately, the Z component – use to identify the binomial time-temperature boundaries to eliminate pathogens microorganism – is much higher for the nutritional components (such as vitamins, minerals among other) than to pathogens microorganisms which can provoke disease and, in some cases, deaths. Therefore, despite there is some nutritional losses in thermal treatments, it is minimal and insignificant when compared to the benefits in safety and health maintenance. In addition, it is important to highlight that this loss also happens at the kitchen (home and restaurants), however, without accurate control of the process parameters – such temperature and time, which can increase the risk of microbial contamination. A minimal mistake in the binomial time-temperature control can result in enhancement of the resistant spores, which can cause foodborne disease. Furthermore, is it possible to compensate the nutritional losses with techniques such as microencapsulation (and others similar), that permit fortifying food by adding nutrients, minerals and nourish components by their chemical interactives and thermodynamic affinities (11, 39, 89, 98, 116).
